# Association of placenta weight and morphology with term low birth weight: A case–control study

**DOI:** 10.1515/med-2025-1264

**Published:** 2025-09-26

**Authors:** Jing Liu, Die Liu, Haixiao Liang, Qi Sun, Yuanmei Chen, Fang Ye, Qi Zhang

**Affiliations:** Department of Pediatrics, China-Japan Friendship Hospital, Beijing, China

**Keywords:** placenta weight, placenta morphology, low birth weight, term birth

## Abstract

**Background and aim:**

Few studies identified the role of the placenta in birth weight. We aimed to explore the connection between placenta weight, morphology, and term low birth weight (TLBW).

**Methods:**

This was a case–control study of neonates born at ≥37 weeks’ gestation enrolled in a general hospital from January 2018 to November 2022. Cases (<2,500 g) identified via birth certificates or medical records were matched with controls on the basis of date of birth, gestational age, sex, and maternal age. A nomogram predictive model was constructed based on logistic regression, using placenta parameters and perinatal information.

**Results:**

A total of 290 neonates (115 with diagnosed TLBW and 175 controls) were determined. There were no significant differences between the two groups concerning gestational age (265.5 ± 5.4 vs 266.3 ± 5.3 days, *P* = 0.1397), gender, and maternal age at delivery (32.0 ± 3.5 vs 32.1 ± 3.1 years, *P* = 0.867). The placenta weight (479.0 ± 80.1 vs 597.1 ± 83.1 cm), length (16.3 ± 2.1 vs 18.8 ± 2.0 cm), width (14.3 ± 2.1 vs 16.9 ± 2.0 cm), and thickness (2.3 ± 0.5 vs 2.4 ± 0.4 cm) in the TLBW group were much lower than those in the control group (all *P* < 0.001). Univariate predictors of TLBW included smaller placental weight, length, width, thickness, volume, and surface area. When put into a multivariate model, placental weight (aOR for per 10 g increase: 0.89; 95% CI: 0.84–0.94) and width (aOR for per 1 cm increase: 0.69; 95% CI: 0.54–0.88) remained to be independent predictors even after controlling for relevant confounders. The odds of TLBW increased when placental weight was below the 50th percentile (aOR: 5.08, 95% CI: 2.59–9.95). Placental width below the 50th percentile was significantly associated with an increased risk of TLBW (aOR: 6.57, 95% CI: 2.73–15.82).

**Conclusions:**

Placental weight and width were found to be associated with TLBW. Further studies focusing on placental function, histology, and pathophysiology are needed to better understand the underlying mechanisms influencing fetal growth and TLBW.

## 1 Introduction

Birth weight, an essential indicator of fetal growth, nutrition, and placenta function, is closely related to perinatal survival and later-life health. In 2015, an estimated 20.5 million babies worldwide were born with low birth weight (LBW, <2,500 g), accounting for 14.6% of all live births [1]. According to the gestational weeks, LBW is distinguished into three categories: preterm LBW, term LBW (TLBW), and postterm LBW. About 91% of LBW infants were born in low- and middle-income countries, and the majority was predominated by TLBW [2]. It was discovered that birth weight has a far greater effect on mortality rates than gestational age [3]. Moreover, LBW has become the second leading cause of perinatal death after preterm birth. Notably, TLBW weighing 1,500–2,500 g had a perinatal mortality rate that was 5–30 times that of normal birth weight infants [3]. Growing evidence suggests that LBW raises the risk of adult disease, especially coronary heart disease, hypertension, and chronic renal disease [4,5].

Factors that may affect fetal growth are manifold and can be divided into different categories: fetal, maternal, uterine-placental, and environmental factors. Additionally, it should be highlighted that TLBW is generally accompanied with intrauterine dystrophy due to abnormal placental circulation. The placenta performs crucial physiological tasks like transport, endocrine, metabolic, and immune functions, serving as a regulatory interface between the mother and the fetus [6,7]. Fetal growth and development are critically dependent on the placental transfer capacity of nutrients, waste products, and other solutes, which is determined by several key components, such as placental size, morphology, transporter abundance and activity, maternal supply ability, and uterine-placental blood flow [8–10]. Indeed, poor placentation, including structurally damaged, abnormal development, insufficiency, dysfunction, etc., is associated with adverse pregnancy outcomes such as miscarriage, pre-eclampsia, intrauterine growth restriction (IUGR), preterm birth, and abnormal birth weight [11–14]. Therefore, it is essential to understand how the placenta develops and functions in order to identify potential targets for preventative or therapeutic interventions to promote child–maternal health.

Studies have demonstrated that the placenta structure and appearance could quantify placenta function to some extent. A reduction in placental volume and surface area, as well as increased thickness, may be markers of placental insufficiency [15]. Additionally, aberrant placental shape may be related to reduced placental efficacy [16]. The associations between placenta morphological measurements and health consequences are also identified in the current literature. Placenta weight is proportional to birth weight to the 0.75 power in term infants [17]. Reduced placenta weight, smaller surface area, or increased placenta thickness-to-volume ratio are observed in fetuses with IUGR [18–20]. Small placental size and uteroplacental malperfusion are more common in term small for gestational age (SGA) than in term appropriate for gestational age (AGA) placentas [21].

Although changes in the absolute or relative placenta size or weight do not necessarily indicate changes in placental function, morphometric measurements of the placenta can provide additional insights into aspects of fetal health and pregnancy outcomes. We hypothesize that differences in placenta development affect placenta function, which in turn influences fetal development, and that variations in placenta morphology are associated with neonatal birth weight. Therefore, we conducted this case–control study in order to fully understand the role of placenta morphological changes in the development of TLBW. In this study, we measured a set of gross morphometric parameters of the placenta, including placental weight, thickness, and inner diameter, and evaluated whether these parameters differed between singleton TLBW infants and normal birth weight infants. We also performed univariate and multivariate logistic regression analysis and drew a nomogram.

## 2 Methods

### 2.1 Study design

**Figure 1:**
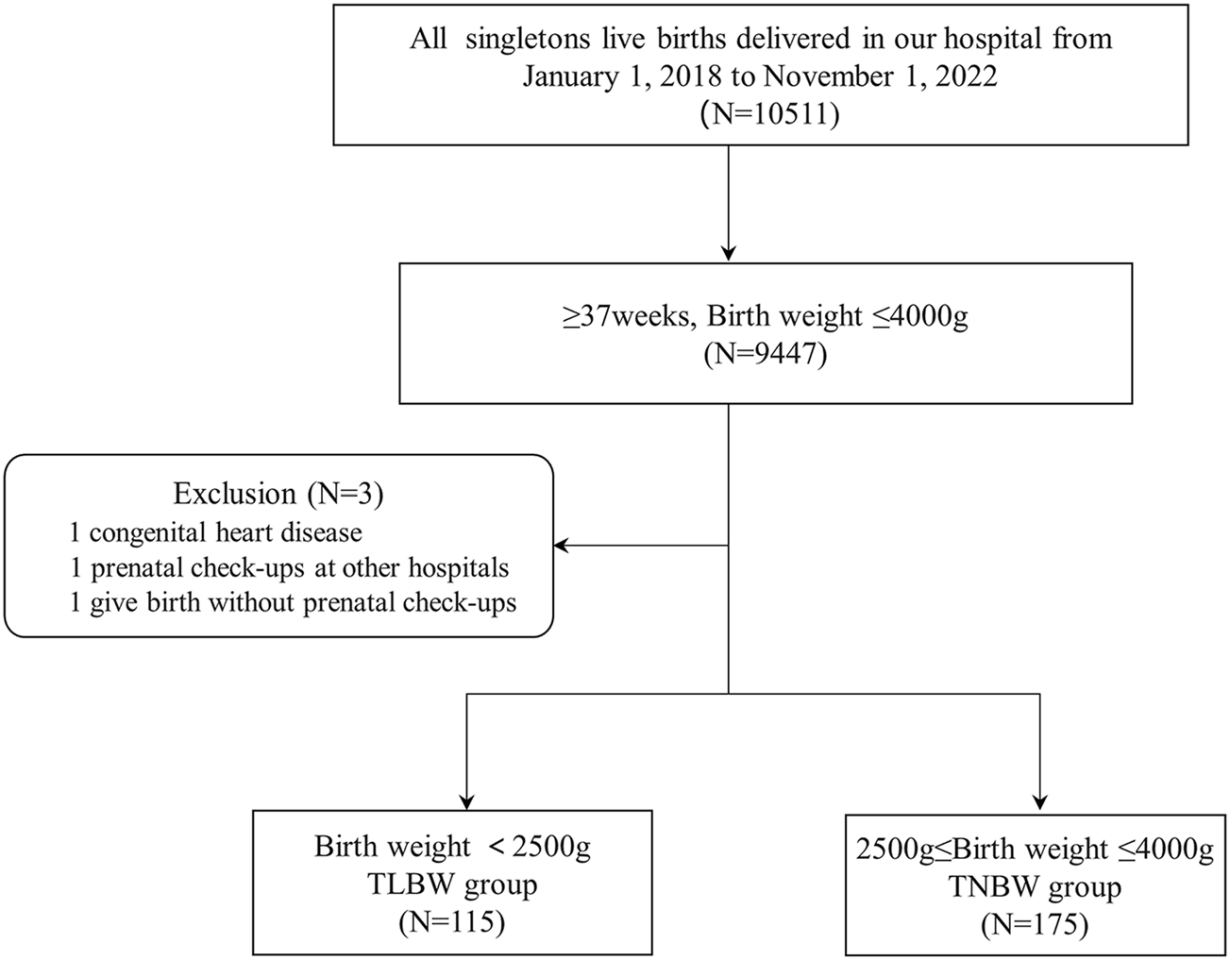
Flow chart of study sample selection.

This case–control study collected the dataset of 10,511 singletons live births delivered in a tertiary general hospital in Beijing from January 1, 2018 to November 1, 2022. We included newborns ≥37 weeks gestational age (GA) with birth weight (BW) <4,000 g. Cases were defined as BW <2,500 g and GA ≥37 weeks with a diagnosis TLBW. We matched each TLBW case with 1.5 term normal birth weight (TNBW) controls based on gestational age, sex, date of birth, and maternal age at childbirth. One infant was excluded due to congenital heart disease and two infants were not included because they either did not receive prenatal check-ups or received care elsewhere finally leaving a final study group of 115 TLBW cases and 175 TNBW controls (Figure 1). The hospital’s Ethical Committee authorized this research. Parental informed consent was obtained in writing from each participant.

### 2.2 Newborn and maternal clinical data

The hospital’s electronic medical record system was reviewed to obtain clinical information regarding both newborns and mothers. We collected neonatal characteristics, such as gender, gestational age, birth weight, gravidity, parity, length, and head circumference. Additionally, we gathered information about each newborn’s mother, including her age at delivery, ethnicity, delivery and conception method, pre-pregnancy weight and height, gestational weight gain, maternal comorbidities, pre-existing chronic diseases, medication use during pregnancy, and any fetal appendage abnormalities. Gestational age was determined based on the last menstrual period, which was cross-checked for accuracy.

### 2.3 Placenta data

Measurements of placental morphological parameters were performed by trained professionals using standardized protocols. After the delivery of the placenta, fetal membranes were trimmed and the umbilical cord was removed within approximately 5 cm of insertion. The excess blood clots were washed away with normal saline, and any water stains on the surface of the placenta were wiped dry with gauze. The following were immediately measured: placental weight was measured using a standard scale with an accuracy of 1 g; the placental length diameter was measured along the longest axis, and the width diameter was measured along the axis perpendicular to the longest axis; placental thickness was measured at the thickest point, with multiple measurements taken to minimize variability and the maximum value used for analysis. The difference in diameters was calculated as the length diameter minus the width diameter. Placenta surface area and volume are calculated according to the following formula: Surface area = *π*/4* length diameter* width diameter; Volumes = 4/3*π* length diameter/2* width diameter/2* thickness/2; Specific surface area (Sv) = Surface area/Volume [22‒25].

The technicians responsible for weighing the placenta and those measuring the infant’s birth weight were separate individuals who did not have access to each other’s results, thereby ensuring that no measurement bias was introduced.

### 2.4 Statistical analyses

Mean and standard deviation (SD) were reported for continuous variables, while integers and proportions were used for categorical variables. Utilizing appropriate statistical analyses, such as the Chi-square test and the Mann–Whitney *U*-test, we analyzed the difference in characteristics between the TLBW and control groups. Medians and quartiles were calculated to describe the distribution of placental morphology measurements. Logistic regression analyses were carried out to examine the association of the placental morphology indices (placental weight, length, width, thickness, etc.) with TLBW. Considering the nonlinear association pattern, all the subjects enrolled were also divided into two groups according to the median of the placental morphology measurements. Univariate analysis was carried out first, followed by multivariate analysis incorporating variables with significant findings in univariate analysis. Collinearity among the variables in the multivariable model was examined using variance inflation factor (VIF), and significant collinearity variables (i.e., surface area and volume, VIF > 10) were excluded. Adjusted odds ratio (aOR) with a 95% confidence interval (CI) was estimated by adjusting for possible confounders, including gravidity, parity, maternal height, and maternal weight before delivery. A nomogram was constructed to predict TLBW probability based on our final adjusted multivariable model. A two-sided *P* < 0.05 was used to declare statistical significance. R software version 4.1.1 was used for all statistical analyses.


**Informed consent:** Parental informed consent was obtained in writing from each participant.
**Ethical approval:** This study was approved by the China–Japan Friendship Hospital ethics committee board (2023-KY-057). All methods were carried out in accordance with relevant guidelines and regulations.

## 3 Results

### 3.1 General characteristics

Tables 1 and 2 summarize the clinical features of the mothers and neonates enrolled in this study, respectively. The neonates’ gestational age (265.5 ± 5.4 vs 266.3 ± 5.3 days, *P* = 0.1397), gender, ethnicity, maternal age (32.0 ± 3.5 vs 32.1 ± 3.1 years, *P* = 0.867), method of delivery, or conception showed no significant difference between TLBW cases and TNBW group. There was a large difference in birth weight between TLBW (2327.0 ± 161.2 g) and TNBW (3081.3 ± 311.0 g) infants, with associated differences in length and head circumference (Table 1).

**Table 1:** Baseline characteristics of eligible neonates (*n* = 290)

Variables	TLBW group (*n* = 115)	TNBW group (*n* = 175)	*P* value
Gestational age (days), mean ± SD	265.5 ± 5.4	266.3 ± 5.3	0.1397
Sex, *n* (%)			0.431
Male	42 (36.5)	72 (41.1)	
Female	73 (63.5)	103 (58.9)	
Birth weight (g), mean ± SD	2327.0 ± 161.2	3081.3 ± 311.0	<0.0001
Gravidity, mean ± SD	1.5 ± 0.8	1.8 ± 1.0	0.0201
Parity, mean ± SD	1.1 ± 0.3	1.4 ± 0.5	<0.0001
Length, mean ± SD	47.0 ± 1.6	49.8 ± 1.2	<0.0001
Head circumference, mean ± SD	32.0 ± 1.3	33.5 ± 1.3	<0.0001

**Table 2:** Descriptive statistics of maternal information, amniotic fluid, umbilical cord, and placenta characteristics

Variables	TLBW group (*n* = 115)	TNBW group (*n* = 175)	*P* value
**Maternal characteristics**			
Age at delivery (years), mean ± SD	32.0 ± 3.5	32.1 ± 3.1	0.867
Ethnicity, *n* (%)			0.174
Han	101 (87.8)	162 (92.6)	
Others*	14 (12.2)	13 (7.4)	
Delivery mode, *n* (%)			0.108
Forceps delivery	5 (4.3)	5 (2.9)	
Spontaneous vaginal delivery	40 (34.8)	82 (46.9)	
Caesarean section	70 (60.9)	88 (50.3)	
Conception method, *n* (%)			0.928
Spontaneously conceived	110 (95.7)	167 (95.4)	
IVF-ET	5 (4.3)	8 (4.6)	
Maternal height, mean ± SD	161.3 ± 5.3	163.1 ± 4.8	0.003
Maternal weight before delivery, mean ± SD	66.6 ± 10.1	70.1 ± 9.1	0.0012
Gestational weight gain (kg), mean ± SD	10.9 ± 4.5	11.6 ± 4.1	0.1867
Pre-pregnancy BMI, mean ± SD	25.6 ± 3.3	26.3 ± 3.4	0.0551
PROM, *n* (%)	27 (23.5)	54 (30.9)	0.171
Preeclampsia, *n* (%)	12 (10.4)	9 (5.1)	0.089
Gestational hypertension, *n* (%)	9 (7.8)	5 (2.9)	0.053
GDM, *n* (%)	32 (27.8)	46 (26.3)	0.772
Gestational anemia, *n* (%)	23 (20.0)	40 (22.9)	0.564
Pregnancy with uterine fibroids, *n* (%)	16 (13.9)	20 (11.4)	0.53
Pregnancy complicated hypothyroidism, *n* (%)	6 (5.2)	13 (7.4)	0.457
GBS (+), *n* (%)	2 (1.7)	5 (2.9)	0.707
Maternal chronic disease before pregnancy, *n* (%)			
Hypothyroidism	6 (5.2)	9 (5.1)	0.978
Connective tissue disease	6 (5.2)	1 (0.6)	0.017
Hashimoto thyroiditis	1 (0.9)	4 (2.3)	0.651
Others^†^	6 (5.2)	11 (6.3)	0.705
Medication use in pregnancy, *n* (%)	20 (17.4)	31 (17.7)	0.944
Levothyroxine	8 (7.0)	18 (10.3)	0.332
Aspirin	5 (4.3)	4 (2.3)	0.491
Labetalol	2 (1.7)	6 (3.4)	0.485
Prednisone	4 (3.5)	1 (0.6)	0.083
Others^§^	2 (1.7)	5 (2.9)	0.707
**Amniotic fluid characteristics**			
Oligohydramnios, *n* (%)	26 (22.6)	13 (7.4)	<0.001
Meconium-stained amniotic fluid, *n* (%)	8 (7.0)	1 (0.6)	0.003
**Umbilical cord characteristics**			
Umbilical cord around the neck, *n* (%)	35 (30.4)	52 (29.7)	0.896
Umbilical cord twist, *n* (%)	26 (22.6)	14 (8.0)	<0.001
Umbilical cord prolapse, *n* (%)	4 (3.5)	2 (1.1)	0.219
True umbilical cord knot, *n* (%)	2 (1.7)	1 (0.6)	0.565
**Placenta characteristics**			
Velamentous placenta, *n* (%)	3 (2.6)	1 (0.6)	0.304
Battledore placenta, *n* (%)	4 (3.5)	7 (4.0)	1
Placental abruption, *n* (%)	2 (1.7)	2 (1.1)	0.65
Placenta adhesion, *n* (%)	2 (1.7)	4 (2.3)	1
Low-lying placenta, *n* (%)	7 (6.1)	17 (9.7)	0.346
Placenta previa, *n* (%)	1 (0.9)	3 (1.7)	1

*Includes Hui, Manchu, Mongolians, and other ethnic minorities.

^†^Includes hyperthyroidism, asthma, nephritis, epilepsy, and others.

^§^Includes insulin, inhaled corticosteroids, and others.

IVF-ET, *in vitro* fertilization and embryo transfer; BMI, body mass index; PROM, premature rupture of membranes; GDM, gestational diabetes mellitus; GBS, group B streptococcus.

We observed that both the maternal weight and weight before delivery were significantly lower in the TLBW group (*P* < 0.05), while gestational weight gain and pre-pregnancy body mass index (BMI) did not show significant differences between the two groups (Table 2). Pregnancy complications (such as premature rupture of membranes (PROM), preeclampsia, gestational diabetes mellitus [GDM], hypertension, anemia, uterine fibroids, and hypothyroidism), some maternal chronic disease before pregnancy (including hypothyroidism and Hashimoto thyroiditis), and medication use in pregnancy (for example, levothyroxine, aspirin, labetalol, and prednisone) did not significantly differ (*P* > 0.05). The TLBW group had a higher incidence of oligohydramnios, meconium-stained amniotic fluid, and umbilical cord twist (*P* < 0.05). However, no significant differences were seen between the two groups with regard to the incidence of umbilical cord around the neck or prolapse, velamentous or battledore placenta, placental abruption or adhesion, etc. (*P* > 0.05).

The mean, interquartile range values of the placental morphology measurements are shown in Table S1. The placenta weight (479.0 ± 80.1 vs 597.1 ± 83.1 cm), length (16.3 ± 2.1 vs 18.8 ± 2.0 cm), width (14.3 ± 2.1 vs 16.9 ± 2.0 cm), and thickness (2.3 ± 0.5 vs 2.4 ± 0.4 cm) in the TLBW group were much lower than those in the control group (all *P* < 0.001).

### 3.2 Placental weight and width are associated with TLBW

**Figure 2:**
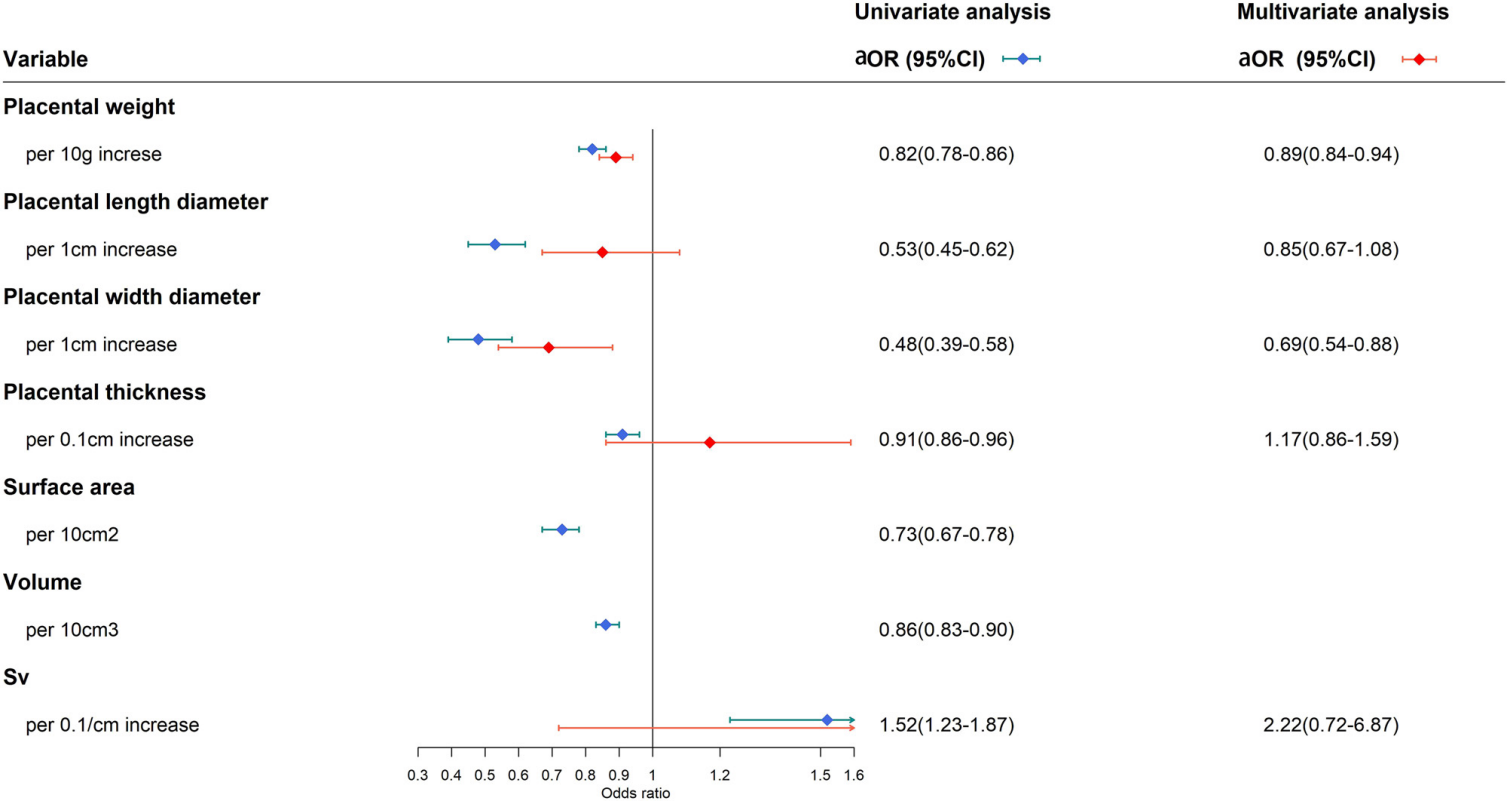
Forest plots of univariate and multivariate logistic regression analysis results of continuous placental morphology measurements.

Univariate predictors of TLBW included placental weight, length, width, thickness, volume, surface area, and specific surface area. Among these, smaller placental weight, length, width, thickness, volume, and surface area were identified as associated with TLBW, while smaller specific surface area was found to be associated with a lower risk of TLBW (Figure 2). Placental weight (aOR for per 10 g increase: 0.89; 95% CI: 0.84–0.94) and width (aOR for per 1 cm increase: 0.69; 95% CI: 0.54–0.88) remained to be independent predictors even after controlling for relevant confounders in the final multivariate regression model (Figure 2).

**Figure 3:**
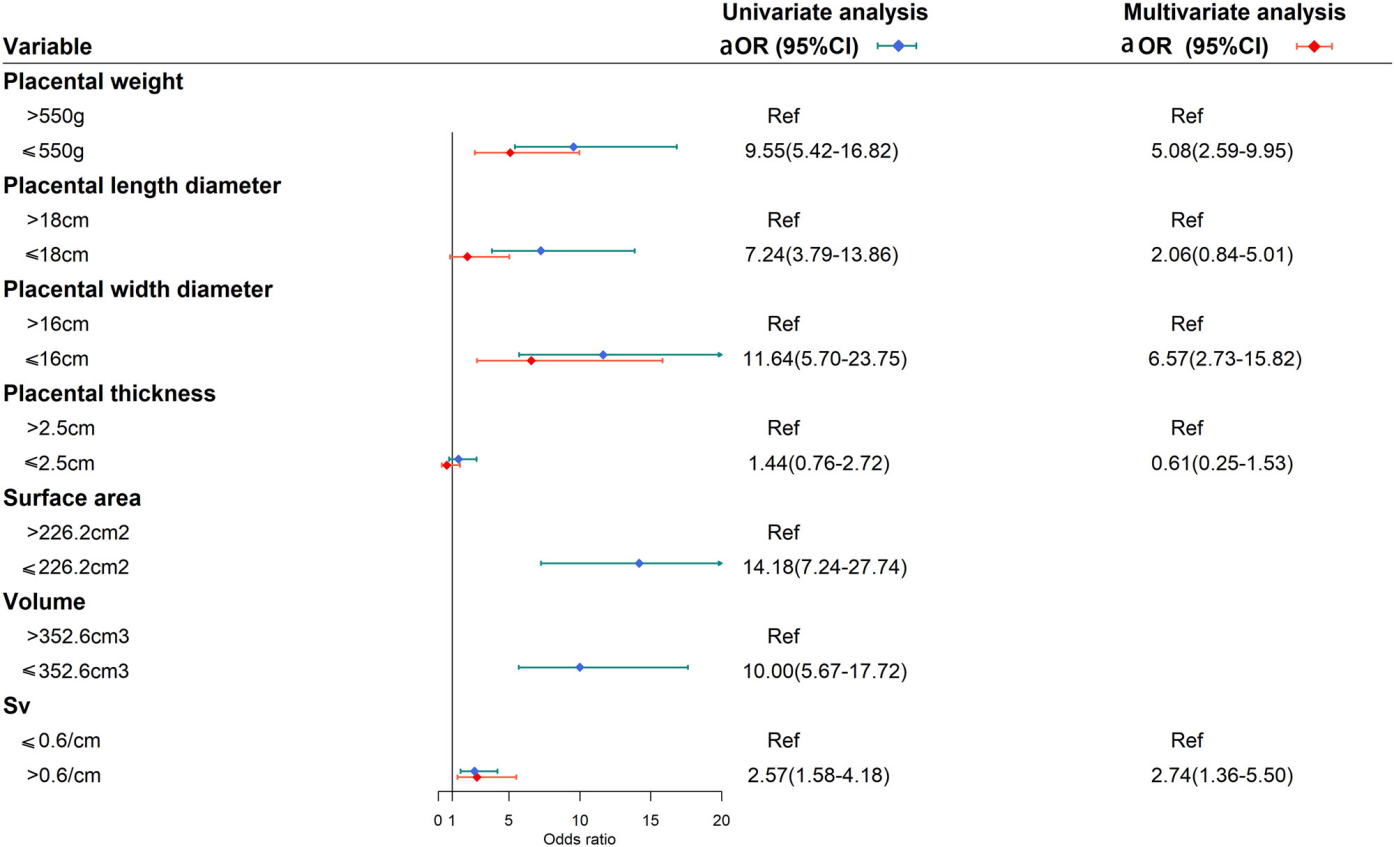
Forest plots of univariate and multivariate logistic regression analysis results of categorical placental morphology measurements.

The results were consistent when these morphometric parameters were analyzed as categorical variables (Figure 3). We observed that the odds of TLBW were higher when placental weight was below the 50th percentile (aOR: 5.08, 95% CI: 2.59–9.95) in the multivariate model. Similarly, placental width below the 50th percentile was significantly associated with an increased risk of TLBW (aOR: 6.57, 95% CI: 2.73–15.82).

**Figure 4:**
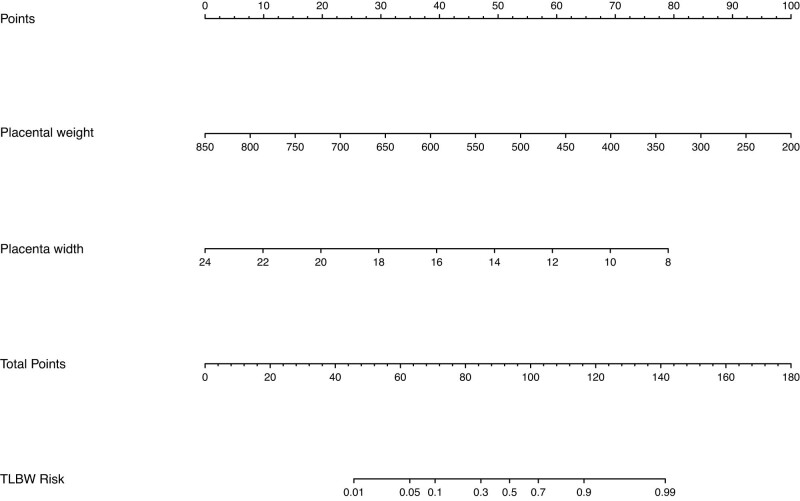
Nomogram for TLBW.

**Figure 5:**
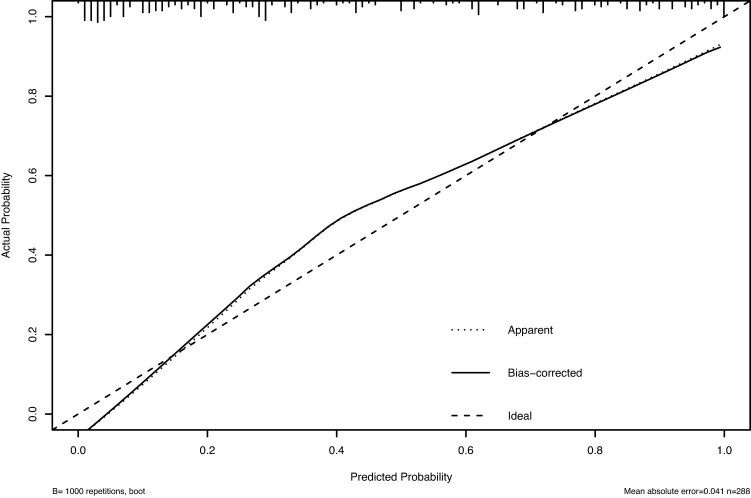
Calibration curves for the nomogram.

**Figure 6:**
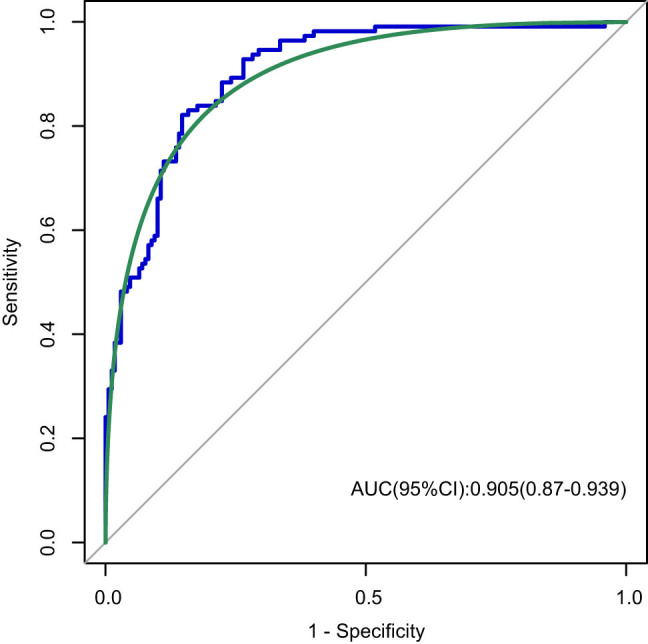
ROC curve of the nomogram.

A nomogram was developed to predict the probability of TLBW based on the two independent parameters of placental weight and width (Figure 4). Each predictor is given a score on the point scale axis based on its value, and the total score is calculated by adding each individual score. The predicted TLBW risk can be determined by projecting the total score line straight to the probability scale line at the bottom. The calibration plots indicated a high level of agreement between the prediction model and the actual results (Figure 5). To further evaluate the performance of the prediction model, a receiver operating characteristic (ROC) curve was plotted (Figure 6). The area under the curve was 0.905 (95% CI: 0.87–0.939), suggesting that the model has good discriminatory ability.

## 4 Discussion

The size, shape, and structure of the placenta are crucial factors for healthy fetal development. There is ongoing research and debate about the relationship between placental morphological changes and adverse pregnancy outcomes. Our case–control matched design study included around 300 term singleton livebirths delivered in a tertiary general hospital. To our knowledge, this study is one of the largest sample size studies conducted to date, and has employed statistical methods such as univariate and multivariate logistic regression to identify predictors of TLBW. We observed that placental weight and width were associated with increased risk of TLBW. The risk of TLBW decreased by 11 and 31% for every 10 g increase in placental weight and 1 cm increase in width diameter, respectively. Neonates with a placental weight less than 550 g had a 5.08-fold higher risk of TLBW compared to those with a placental weight greater than 550 g. Placental width diameter ≤16 cm showed a 6.57-fold of TLBW compared with those with longer width (more than 16 cm).

In the present study, we identified significant differences in placental weight between the TLBW and control groups. Similar findings were reported in previous studies, including one by Zheng et al., which found a significant difference in placental weight between TLBW and TNBW infants, with the TLBW group having a significantly lower placental weight (492.8 ± 38.5 g) compared to the TNBW group (701.2 ± 106.7 g) [26]. In addition, a study in India demonstrated that preterm LBW had lower mean placental weight than TLBW infants (410.2 g ± 101.3 vs 469.1 ± 55.8 g) [27]. Similar results were found in SGA and neonates with IUGR. Biswas and Ghosh reported a mean placental weight of 333.32 ± 75.59 g for the IUGR group and 416.77 ± 63.03 g for the control group [18]. Chisholm and Folkins observed that the mean placental weight of term SGA group was 351.85 ± 139.28 g and that of AGA group was 445.90 ± 86.33 g [21].

More importantly, our logistic regression analysis further confirmed that placental weight was an independent predictive factor of TLBW. There was a significant relationship between placental weight and TLBW whether treated as a continuous or categorical variable. Many studies have shown that there is a positive correlation between placental weight and birth weight. For example, the prospective study conducted in Mexico found that for each 1 g increase in placental weight, there was a corresponding increase of approximately 1.98 g in birth weight among term newborns [28]. Similarly, placenta weight percentile curves according to gestational weight for singleton and twins deliveries in Canadian population fit by appearance positive linear relations [29]. These findings suggest that placental weight may play an important role in fetal growth and development, as well as serve as an indicator of efficacy in nutrient and oxygen transport. Additionally, the ratio of birth weight to placental weight has been proposed as a marker of placental efficiency [30–32].

Decreased placental width diameter was another predictor of TLBW in this study. Although length, thickness, volume, surface area, and specific surface area were significant on univariate analysis, they failed to confirm the multivariate importance. Haeussner et al. found that the shape of the placental disc converges toward a roundish, but not precisely circular [33]. Lower birth weight was observed in non-round/oval placentas [34]. The difference in length and width reflects the degree of ovality of the placenta. In this study, however, the difference in diameters between the two groups was not significant. We speculate that differences in placental shape may represent an adaptation to the unique environmental conditions experienced by each fetus, which may differ from placental weight.

Indeed, in addition to placental weight and gross morphological parameters, placental parameters measured by ultrasound, such as placental volume, thickness, and vascularity, have been shown to correlate with fetal growth. A prospective study of 619 Chinese women with singleton pregnancy revealed that placental volume in early pregnancy was an independent predictor of SGA [35]. Studies have demonstrate that placental evaluation using three-dimensional ultrasonographic placental measurements at 11–14 weeks of gestation can significantly improve the early prediction of SGA [36,37]. However, there is currently no standardized method for assessing antepartum placental growth during pregnancy. Additionally, the additional time required for 3D measurements compared to 2D measurements, the lack of machines capable of multiplanar reconstruction, and the lack of a universal reference range for placental measurements are some of the limitations that must be addressed for ultrasound measurement of the placenta to become a standard diagnostic tool.

Overall, this study has several strengths, including a sufficient sample size and the use of reproducible and reliable measurement indicators for the general parameters of the placenta. Furthermore, we identified two important predictors (placental weight and placental width) that are important parameters for prediction of TLBW. We also controlled for confounding factors such as gestational age, mother’s height, and weight before delivery, which strengthens the validity of our results. However, there were several limitations. First, we only measured placental morphology at delivery, and future studies that include parameters related to placental structure and function during pregnancy may offer more valuable insights. Second, this study was conducted at a single center, which may limit the generalizability of our results. Multicentric validation could enhance the external applicability of our findings. Third, although matching was performed on key variables, other important determinants of fetal growth were not accounted for *a priori*, and residual confounding remains possible despite *post hoc* adjustment.

## 5 Conclusions

In conclusion, it appears that the placentas in the TLBW group were generally smaller than those in the TNBW group, as evidenced by differences in weight, length, width, thickness, volume, and surface area. We identified placental weight and width as independent predictors of TLBW, indicating that both placental weight and size or shape may be linked to fetal growth. However, as this study is observational, further research is needed to better understand the underlying mechanisms and to explore effective strategies for monitoring placental health during pregnancy. Future studies should also include antepartum imaging or biomarkers to enhance prenatal prediction and assess the clinical applicability of these findings.

## Supplementary Material

Supplementary Table
