# Supplementary material for: Association of placenta weight and morphology with term low birth weight: A case–control study
Source: Open Med (Wars). 2025 Sep 26;20(1):20251264. doi: 10.1515/med-2025-1264 (PMC12487766; doi:10.1515/med-2025-1264)
Supplement: Supplementary Table [file med-2025-1264-sm.pdf]

Supplementary material

Table S1: Summary of placental morphology measurements

Variables	Group	Mean	SD	Min	P25	P50	P75	Max
Placental weight (g)*	TLBW	479.0	80.1	200	430	**500**	540	690
	TNBW	597.1	83.1	400	540	**600**	650	830
	Total	550.3	100.2	200	500	**550**	610	830
Placental length diameter (cm)*	TLBW	16.3	2.1	11	15	**16**	18	25
	TNBW	18.8	2.0	14	18	**18**	20	25
	Total	17.8	2.4	11	16	**18**	20	25
Placental width diameter (cm)*	TLBW	14.3	2.1	9	13	**14**	16	20
	TNBW	16.9	2.0	10	16	**17**	18	24
	Total	15.8	2.4	9	14	**16**	18	24
Placental thickness (cm)*	TLBW	2.3	0.5	1	2	**2**	2.5	5
	TNBW	2.4	0.4	1.5	2	**2.5**	2.5	4
	Total	2.4	0.5	1	2	**2.5**	2.5	5
Surface area (cm^2)*	TLBW	184.6	46.6	86.4	153.2	**176.7**	212.1	392.7
	TNBW	251.1	53.4	125.7	226.2	**251.3**	282.7	471.2
	Total	224.8	60.3	86.4	179.1	**226.2**	254.5	471.2
Volume (cm^3)*	TLBW	277.6	86.8	94.2	217.8	**274.9**	333.8	552.9
	TNBW	412.7	121.8	204.2	335.1	**392.7**	471.2	942.5
	Total	359.1	127.6	94.2	268.1	**352.6**	424.1	942.5
Difference in diameters (cm)	TLBW	2.0	1.8	0	1	**2**	2	10
	TNBW	1.9	1.5	0	1	**2**	2	9
	Total	1.9	1.6	0	1	**2**	2	10
Sv (1/cm)*	TLBW	0.70	0.18	0.3	0.6	**0.75**	0.75	1.5
	TNBW	0.63	0.10	0.375	0.6	**0.6**	0.75	1
	Total	0.66	0.14	0.3	0.6	**0.6**	0.75	1.5

*Represents difference (*P* value <0.001) between the means of two group.

Sv, specific surface area.
